# Biomechanical behavior of different designs of hybrid abutment-restoration on the posterior crown: a finite element analysis

**DOI:** 10.1590/0103-6440202305539

**Published:** 2023-12-22

**Authors:** Adna Alves Rocha, Marco Aurélio de Carvalho, Dimorvan Bordin, Altair Antoninha Del Bel Cury, Priscilla Cardoso Lazari-Carvalho

**Affiliations:** 1 School of Dentistry, Evangelical University of Goias (UniEVANGÉLICA), Anápolis, Brazil.; 2 Department of Oral Rehabilitation, School of Dentistry, Evangelical University of Goias (UniEVANGÉLICA), Anápolis, Brazil.; 3 School of Dentistry, Universus Veritas UNG, Guarulhos, SP Brazil.; 4 Universidade São Judas Tadeu, São Paulo, SP, Brazil.; 5 Department of Prosthodontics and Periodontology, Piracicaba Dental School, State University of Campinas Brazil.; 6 Department of Oral Rehabilitation, School of Dentistry, Evangelical University of Goias (UniEVANGÉLICA), Anápolis, Brazil.

**Keywords:** dental implants, finite element analysis, CAD/CAM

## Abstract

This study aimed to evaluate the influence of material and crown design on the biomechanical behavior of implant-supported crowns with hybrid abutment (HA) through three-dimensional (3D) finite element analysis. The study factors were the type of material used as the mesostructure or crown (zirconia, lithium disilicate, and hybrid ceramic) and the crown design cemented to the titanium base (mesostructure cemented to the titanium base and a crown cemented on it (HaC); hybrid crown-abutment, the abutment and crown are manufactured as a single piece and cemented to the titanium base (HC); monolithic crown cemented on the titanium base and screwed to the implant (CS); and monolithic crown cemented on the titanium base (CC). Four 3D models were constructed using an implant with an internal connection, and an oblique load of 130 N was applied at 45° to the long axis of the implant. The models were evaluated using the von Mises stress for crown, abutment, screw, and implant and maximum principal stress for bone tissues. The lowest stresses occurred in the groups with a lower elastic modulus material, mainly hybrid ceramics, considered a material with greater resilience. The cemented crown group presented the lowest stress values. The stresses were concentrated in the cervical region of the crown at the titanium crown/base interface. Mesostructures made of materials with a higher elastic modulus exhibited a higher concentration of stress. The presence of a screw hole increased the stress concentration in the ceramic crown. Cemented ceramic crowns exhibited better biomechanical behavior than screw-retained crowns.

## Resumo

O estudo teve como objetivo avaliar a influência do material e do desenho da coroa no comportamento biomecânico de coroas implanto-suportadas com pilar híbrido (AH) por meio da análise tridimensional (3D) de elementos finitos. Os fatores de estudo foram o tipo de material do HA ou coroa (zircônia, dissilicato de lítio e cerâmica híbrida) e o desenho da coroa sobre a base de titânio (mesoestrutura cimentada à base de titânio e coroa total cimentada em cerâmica; pilar e coroa fabricados em peça única cimentada à base de titânio; coroa monolítica cimentada sobre a base de titânio e aparafusada ao implante; e coroa monolítica cimentada à base de titânio). Quatro modelos 3D foram construídos e uma carga oblíqua de 130 N foi aplicada a 45° em relação ao longo eixo do dente. Os modelos foram avaliados utilizando a tensão de von Mises para a coroa, pilar, parafuso, implante e tensão principal máxima (tensão de tração) para o tecido ósseo. As menores tensões ocorreram nos grupos que apresentaram um material com menor módulo de elasticidade, principalmente a cerâmica híbrida, considerada um material com maior resiliência. Com relação às diferentes configurações, o modelo com coroa cimentada apresentou os menores valores de tensão. As tensões foram concentradas na região cervical da coroa na interface entre coroa e base de titânio. Mesoestruturas feitas de materiais com maior módulo de elasticidade exibiram maior concentração de tensões. A presença de um orifício para parafuso aumentou a concentração de tensão na coroa de cerâmica. As coroas cerâmicas cimentadas exibiram melhor comportamento biomecânico do que as coroas parafusadas.

## Introduction

The success of implant restorations depends not only on osseointegration but also on achieving the natural and harmonious appearance of missing teeth while restoring their function. Titanium abutments associated with metal-ceramic crowns are the standard treatment in implant dentistry, with high survival rates^1^ ^,^ ^2^. However, titanium is a metallic substrate and consequently may provide an esthetic unpleased graying effect on the peri-implant marginal mucosa^3^.

In addition to esthetic demands, digital workflows associated with ceramic materials offer patients a metal-free restoration alternative. Owing to its well-documented high fracture strength, good esthetics, and superior biocompatibility, zirconia (Zr) has attracted significant interest for use as an implant abutment^4^ ^,^ ^5^. The esthetic benefits of ceramic abutments over metal abutments have been well documented in clinical and in vitro studies, although their mechanical performance remains a matter of concern since recent studies of Zr abutments with external connections or internal connections have revealed lower fracture resistance than titanium abutments, especially with internal connections^6^ ^,^ ^7^ ^,^ ^8^.

Hybrid abutments (HAs) have emerged as alternatives to implant-supported crowns. The HA, as it is known, consists of two parts: a ceramic mesostructure cemented on a titanium base. The first improves the esthetics of the peri-implant mucosa, and the second is responsible for maintaining the titanium-to-titanium connection^9^. Studies have shown that the use of a titanium base provides better support for ceramics and accurate fit with the implant and improves abutment fracture resistance ^8^ ^,^ ^9^ ^,^ ^10^. Its use avoids the weakest point of the Zr abutment in the implant-abutment contact area. The most common materials used are the Zr HAs, as they exhibit a higher bending moment strength than Zr-only abutments^11^ ^,^ ^12^. In summary, Zr abutments with a titanium base have a high survival rate and show no difference from metal abutments after a mean observation period of 5-7 years^5^.

Despite its success rate, Zr has biomechanical properties that are significantly different from those of natural teeth. Esthetics, strength, and biology must be carefully balanced to create an ideal restorative abutment for implant-supported restorations, with the ultimate goal being to emulate the natural tooth and its ability to distribute occlusal forces, especially in implants, where the periodontal ligament is missing^13^. Another material option used is lithium disilicate (Ld) ceramics, which have also proven to be a successful esthetic option compared with Zr. The use of Ld as a mesostructure structure is relevant in this context. Although this material does not have sufficient mechanical strength to connect directly to the implant platform, its use seems suitable when combined with a metallic base^8^ ^,^ ^14^. However, there are few reports in the literature regarding their use in Has^13^ ^,^ ^15^ ^,^ ^16^.

Additional materials have been investigated for their physical-mechanical properties similar to those of natural dental tissues. New-generation blocks for computer-aided design and computer-aided manufacturing (CAD-CAM) processing have been developed, such as hybrid ceramics. This material has abrasion resistance, high flexural strength, and elasticity similar to dentin, and its wear is comparable with that of common dental ceramics, whereas the wear of the antagonist tooth is lower than that of conventional ceramics^17^. In addition, it has a Weibull modulus, friability, and high strength, indicating its use in implant-supported restorations^17^. However, its use in association with HAs has not yet been proven.

There are two possibilities for HA setup: as an HA cemented to the titanium base and a cement-retained all-ceramic crown or as a hybrid crown-abutment, where the abutment and crown are manufactured as a single piece cemented to the titanium base; later, it is screwed to the implant. For the last option, the crown must contain a central hole to allow the connection between the titanium base and screw access to the implant. The presence of a hole in the crown can reduce resistance^8^; however, this statement remains controversial^18^.

Although the use of HAs is considered a promising solution, there is little data on the biomechanical behavior of screw-retained molar restorations based on Zr, Ld, and hybrid ceramics^5^. Therefore, this study aimed to evaluate the influence of material and crown configuration in posterior teeth on the biomechanical behavior of implant-supported restorations through three-dimensional (3D) finite element analysis. This study hypothesized that the material and crown designs influence the biomechanical behavior of implant-supported posterior crowns.

## Materials and methods

The present study was an in-silico study using a 3D finite element method. The study factors were the type of material used as the mesostructure or crown (Zr, Ld, and hybrid ceramic) and the crown design cemented to the titanium base (HA cemented to the titanium base and on top of it a full ceramic cemented crown [HaC]; hybrid crown-abutment, where the abutment and crown are manufactured as a single piece and cemented to the titanium base and screwed to the implant [HC]; monolithic crown cemented on the titanium base and screwed to the implant [CS]; and monolithic crown cemented on the titanium base [CC]). The results were analyzed using the von Mises stress for crown, abutment, screw, and implant and maximum principal stress (tensile stress) for bone tissue.

**Figure 1:**
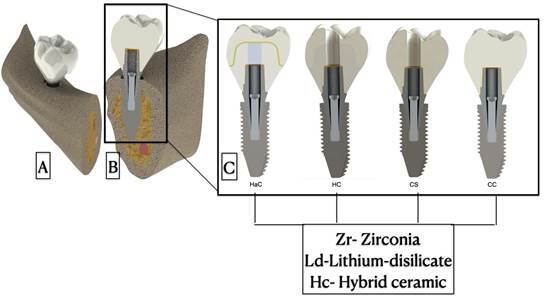
Three-dimensional models of the posterior ceramic crown on morse taper implants inserted in a posterior segment of the mandible. (A) Buccal view and (B) longitudinal section and internal structures. (C) Study groups: HaC, hybrid abutment cemented to the titanium base and on top of it a full ceramic cemented crown; HC, hybrid crown-abutment, where the abutment and crown are manufactured as a single piece and cemented to the titanium base and screwed to the implant; CS, monolithic crown cemented on the titanium base and screwed to the implant; and CC, monolithic crown cemented on titanium base.

A 3D model of the mandibular molar was reproduced based on computed tomographic images (ORTHOPANTOMOGRAPH™ OP300; Instrumentarium Dental™, Finland). Three-dimensional reconstruction of these tomographic images on solid devices for stereolithography (STL) file formats was performed using InVesalius software (version 3.0, 64 bits; Centro de Tecnologia da Informação Renato Archer, Campinas, Brazil) based on a previous study^19^. The STL image files were imported into SolidWorks 2018 CAD software (SOLIDWORKS Corp., MA, USA), and 3D models were generated with the following crown dimensions: 11.5-mm length and 8-mm buccolingual distance. The titanium base, screw, and implant followed the measurements obtained by the manufacturer (titanium base, 4.5 × 4 mm; implant, 4.75 × 11.0 mm). The implant was inserted into the posterior segment of the mandible with the following dimensions: 2 mm-thick cortical bone and 21 mm-high medullary bone, presenting 1 mm infra-bone insertion. Using CAD software and the main model of the first mandibular molar crown, the boolean operations were performed, and four 3D models were generated, as shown in Figure 1. The mesostructure or crowns were luted with a 0.5 mm-thick cement layer. The models were exported to the ANSYS Workbench 19 software (Ansys Inc., Canonsburg, PA, USA) for finite element mesh generation and numerical analysis. The interface between the structures was considered a perfect union, simulating complete osseointegration (bonded contact) at the bone/implant interface. All structures were considered isotropic, homogeneous, and linearly elastic. The mechanical properties (modulus of elasticity or Young’s modulus and Poisson’s ratio) of these structures were taken from the literature to standardize these data and facilitate the comparison of results with those of other studies (Table 1).

**Table 1:** Mechanical properties of the materials according to previous studies.

Structure	Young’s modulus (E) (GPa)	Poisson ratio (δ)	Reference
Cortical bone	13.6	0.26	^2^
Trabecular bone	1.36	0.31	^2^
Titanium	110	0.25	^2^
Zirconia	205	0.22	^3^
Lithium disilicate	96	0.23	^4^
Hybrid ceramic	34.7	0.28	^5^
Feldspatic ceramic	90	0.33	^6^
Resin cement	18.3	0.33	^6^

**Figure 2:**
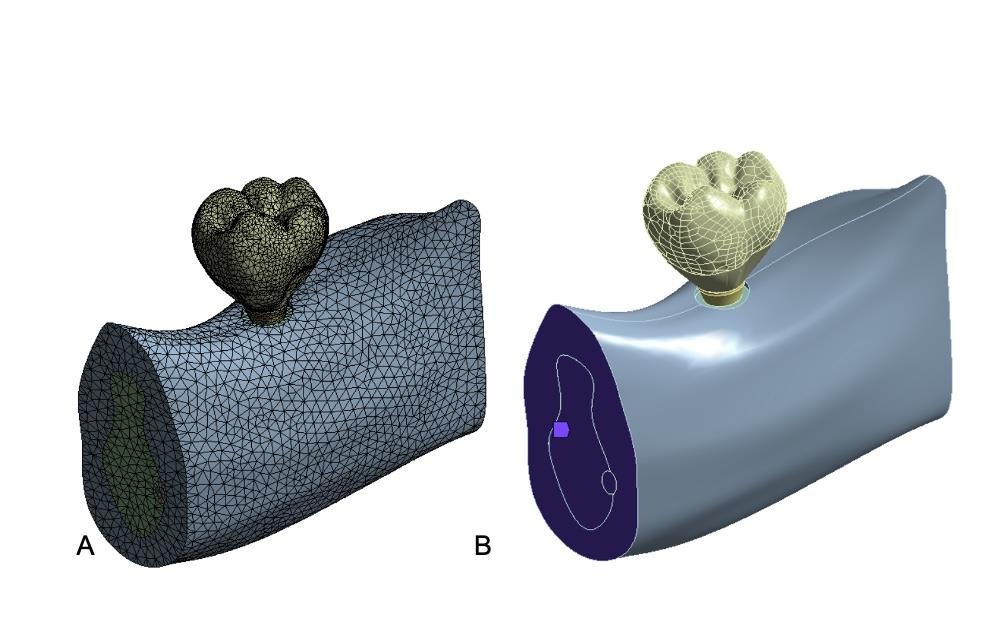
Boundary conditions

The mesh elements were chosen to be the quadratic tetrahedral type, and their size was set to 0.5 mm after 5% analysis convergence^20^ (Figure 2A). Boundary conditions were established on the external surfaces of the modeled bone in all directions (Figure 2B). An oblique load of 130 N was applied at an angle of 45° to the long axis of the tooth^21^.

The results were evaluated following a qualitative analysis of stress distribution and peak concentration and quantitative analysis using the von Mises stress for the crown, abutment, screw, and implant and maximum principal stress (tensile stress) for bone tissues.

## Results

### Titanium Base

**Figure 3:**
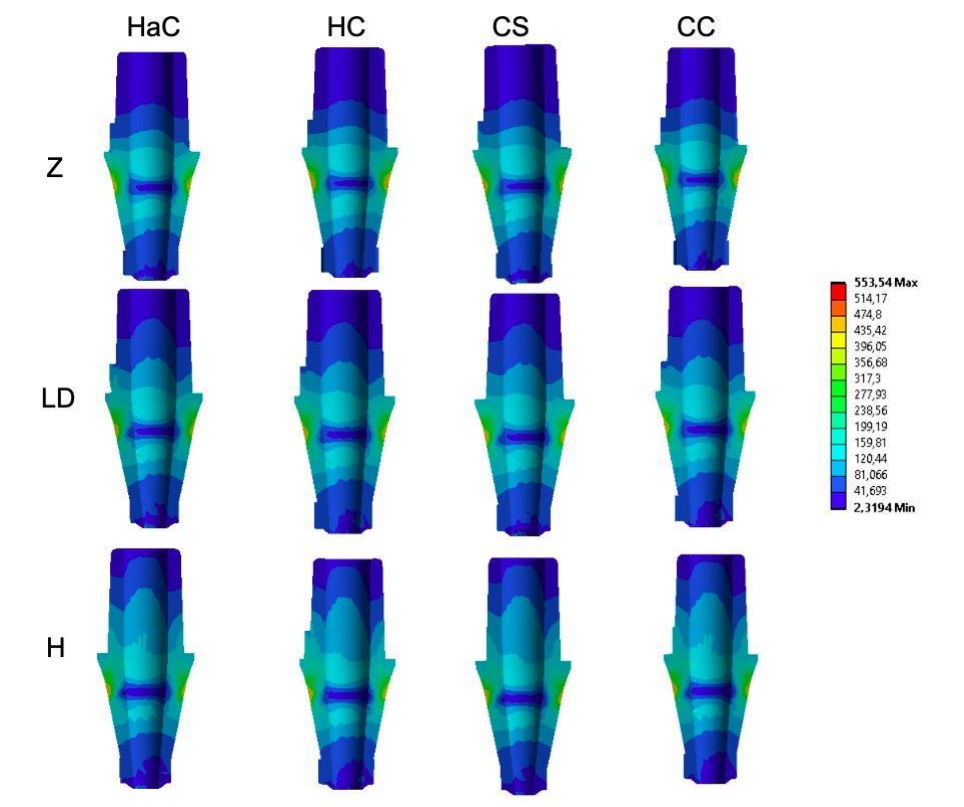
Stress concentration in the titanium base. The stresses are concentrated at the abutment/implant interface.

Table 2 presents the quantitative results. The stresses of the titanium base showed similar stress distributions, where the maximum stress was found in HaC (554 MPa), and the lowest was found in CC-LD (500 MPa). The lowest stress concentrations were observed in the groups that presented a crown or mesostructure of the material with a lower elastic modulus (Ld and hybrid ceramic). Stresses were concentrated at the abutment/implant interface (Figure 3).

**Table 2:** Quantitative results: maximum principal stress for bone tissue and von Mises stress for the implant, screw, crown, and resin cement

Groups		Cortical bone	Trabecular bone	Implant	Titanium base	Screw	Cement	Crown
HaC	Zr	37	16	490	554	70	124	968
	Ld	37	16	490	517	70	136	697
	H	37	16	493	500	70	156	336
HC	Zr	37	15	495	544	81	106	1,240
	Ld	37	15	496	504	81	128	714
	H	38	15	500	507	81	161	314
CS	Zr	38	16	499	528	81	124	1,093
	Ld	37	15	497	505	81	126	632
	H	38	15	500	508	81	119	290
CC	Zr	37	16	492	521	79	116	794
	Ld	37	16	493	500	79	119	464
	H	37	16	495	503	79	117	221

HaC, hybrid abutment cemented to the titanium base and on top of it a full ceramic cemented crown; HC, hybrid crown-abutment, where the abutment and crown are manufactured as a single piece and cemented to the titanium base and screwed to the implant; CS, monolithic crown cemented on the titanium base and screwed to the implant; CC, monolithic crown cemented on the titanium base; Zr, zirconia; Ld, lithium disilicate; H, hybrid ceramic.

### Crown

**Figure 4:**
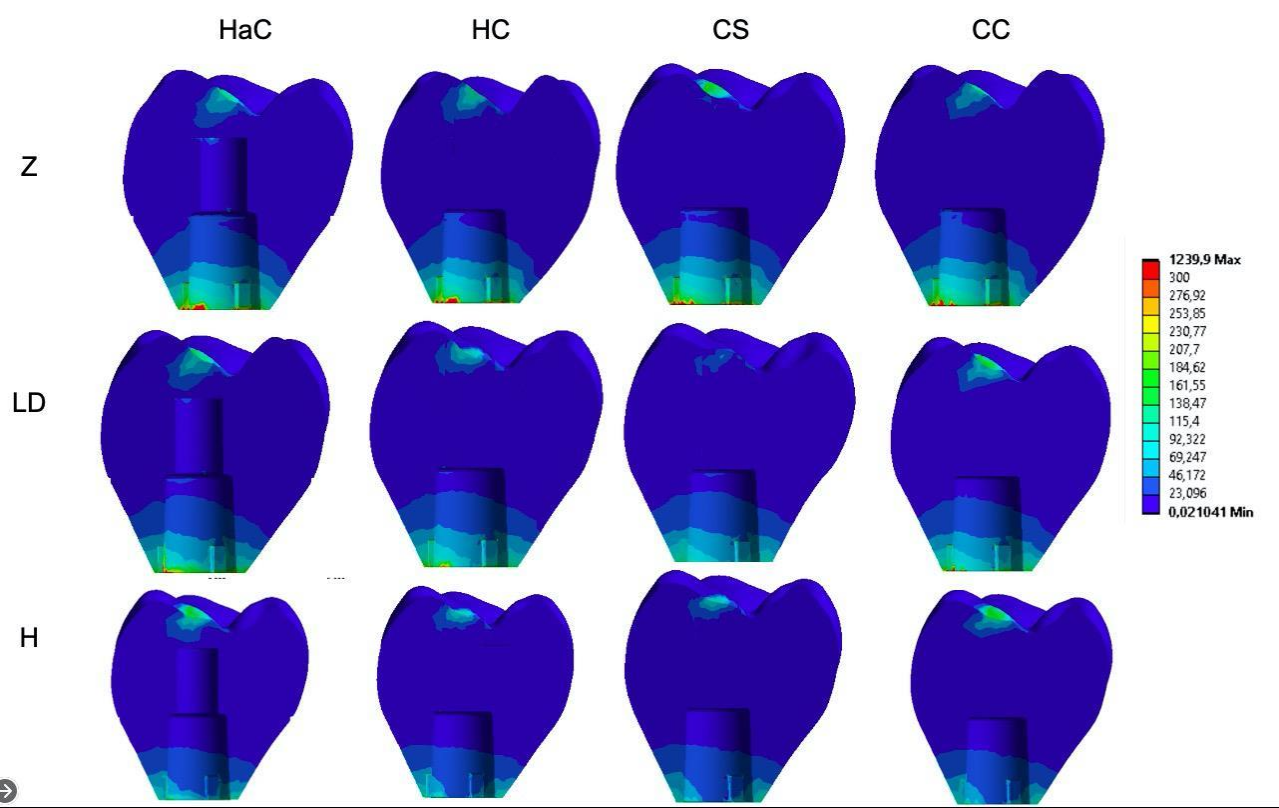
Crown stress concentration pattern. The stresses are concentrated in the cervical region of the crown at the interface between the crown and the titanium base.

The stress values showed a great variety of results between the groups, with stresses between 221 and 1,024 MPa (Table 2). The lowest values occurred in the groups that presented the material with a lower elastic modulus, mainly hybrid ceramics, considered a material with greater resilience (H). Concerning the different configurations, the CC group (cemented crown) presented the lowest stress values. The stresses were concentrated in the cervical region of the crown, at the titanium base/crown interface (Figure 4).

### Ceramic Crown Versus Mesostructure

**Figure 5:**
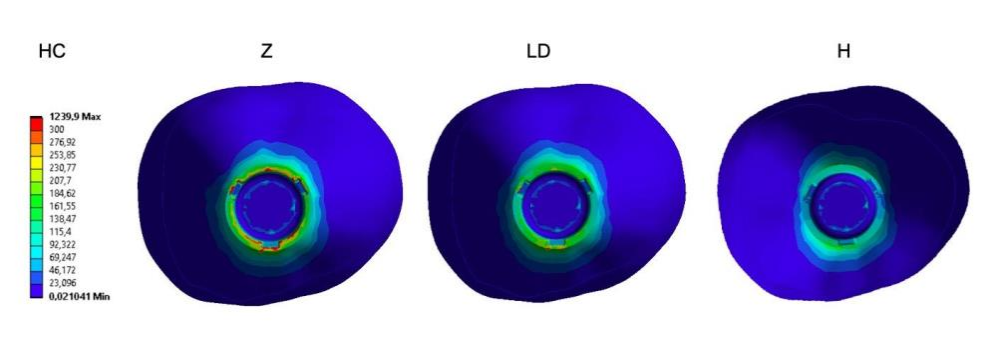
Bottom view of the hybrid crown-abutment group for better visualization of the places with the highest stress peaks.

**Figure 6:**
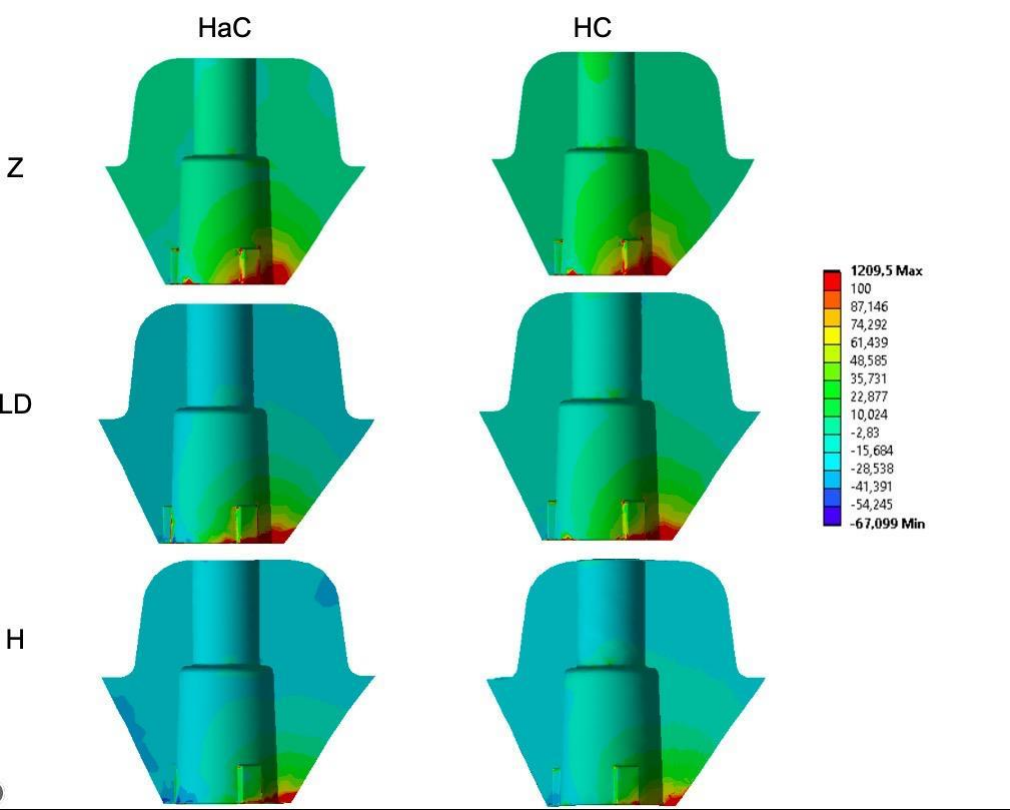
Mesostructure stress concentration pattern. The stresses are concentrated in the cervical region of the crown at the interface between the mesostructure and titanium base.

The results for the crown were also explored to observe whether changing the mesostructure material could modify the stress concentration in the mesostructure/ceramic covering crowns. For this, the results of the HaC and HC groups that presented mesostructures and ceramic coverings were evaluated separately. Similar stress patterns were observed both quantitatively and qualitatively (Figure 5). The highest stress values for the mesostructure were observed in the models with the Zr mesostructure, followed by those with Ld and hybrid ceramics. When comparing the type of crown configuration, those with veneering ceramics cemented over the mesostructure had reduced stress concentrations in the mesostructure (Figure 6).

**Figure 7:**
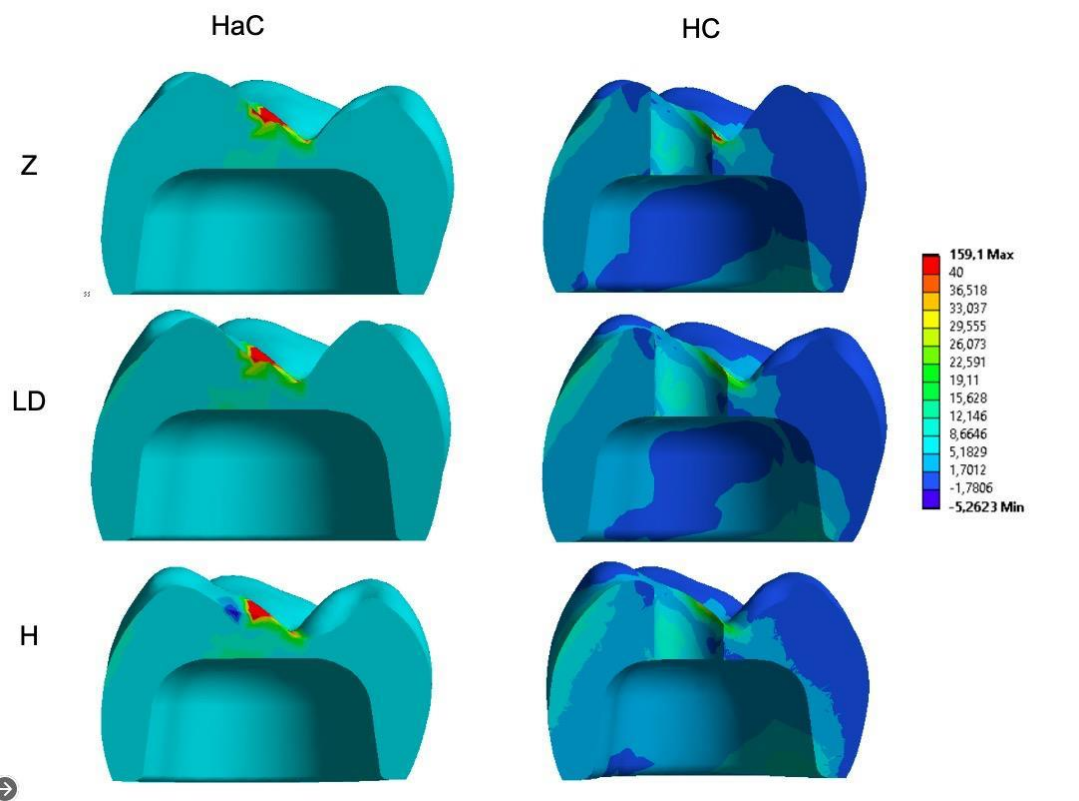
Stress concentration pattern in the ceramic veneer. The stresses are concentrated in the occlusal load application region.

The stress values or veneering ceramics did not change when the mesostructure materials were modified. However, the crown configuration influenced the stress, and the covering ceramics cemented on the mesostructure presented the lowest stress values (Figure 7). The stresses are concentrated in the cervical region of the crown at the interface between the mesostructure and titanium base.

### Implant and Bone Tissue

No changes were observed in the stress values of the trabecular or cortical bone, implants, or prosthetic screws. For the bone tissue, the stresses were concentrated on the lingual aspect of the cortical bone at the implant/bone interface. For implants and prosthetic screws, the stresses were concentrated on the internal face of the implant located in the cervical region, the interface between the implant and the titanium base.

## Discussion

This study aimed to evaluate the influence of material and crown design on the biomechanical behavior of posterior implant-supported restorations. The study hypothesis was confirmed because there were differences in the biomechanical behavior of implant-supported posterior crowns using a titanium base when the material type and crown configuration were modified.

The use of metal bases as an interface with the implant platform has improved the fracture resistance of Zr abutments; however, its strength is still lower than all-metal abutments^22^. Therefore, the combination of crown and HA (ceramic mesostructure cemented on a titanium base) avoids overloading stress concentration in the titanium base^23^. In the present study, it was observed that the different materials used in the mesostructure, despite having widespread mechanical properties, were not able to significantly change the stress generated in the titanium base. The Zr mesostructure merely increased the stress in the titanium base by 11% compared with the other materials. The data showed that the use of different materials or prosthetic designs did not negatively affect the biomechanical performance of titanium bases, indicating their use as a protective interface against plastic deformation at the implant platform^9^.

The results for the mesostructure material showed that Zr mesostructures presented the highest stress concentration, which corroborates the results of the study by Tribst. et al.^24^. However, the literature points to fracture load values greater than 3,000 N, significantly higher than the forces originating from occlusal forces^25^.

HAs made of Ld mesostructures exhibit promising durability and strength after long-term dynamic loading^8^. In the present study, the mesostructures made with Ld and hybrid ceramics exhibited lower stress concentrations than those made with Zr. The results demonstrated that the lower the material resilience, the lower the tensile stress generated at the cervical region of the mesostructure/titanium interface. Therefore, a flexible mesostructure appears interesting^23^. However, such results should be interpreted with caution when comparing hybrid ceramic mesostructures because the hybrid ceramic mesostructure had the lowest fracture resistance values in the anterior teeth^26^.

The present study evaluated four possible restorative solutions combining different abutments and ceramic materials, each with different advantages. Although cement-retained crowns generally exhibit better stress values, the screw access hole in screw-retained crowns is important for reversibility. However, literature regarding the strength of these crowns is controversial^18^. Some studies have suggested that the screw hole decreases the fracture resistance of the crown^27^ which corroborates with the obtained data. Analyzing the screw-retained crowns, the access hole to the screw sealed with composite resin increased the stress generated in the cervical region in contact with the metallic base. The greatest differences between the screw-retained and cement-retained crowns were found in the Zr and Ld models, with the hybrid material showing similar stress between the cemented and screw-retained crowns.

In the present study, the CC group simulated the use of a monolithic crown cemented over a titanium base as if the base was a conventional abutment (universal abutment). Although this group presented lower stress values than the HaC group, it is important to highlight that this restoration modality did not follow the manufacturer’s recommendation. The titanium base is not indicated as a conventional abutment because of the height of the platform, which does not allow the complete removal of the resin cement from the cervical region in an intraoral environment. The group was created for comparison to answer questions regarding the presence of a screw hole for monolithic crowns on a titanium base.

Bilayered ceramic restorations (mesostructure + veneering ceramic) have a higher risk of complications, such as chipping, than monolithic restorations. The shock-absorbing effects of the masticatory loading can explain this. However, it is important to consider that fracture loads using crowns with Zr mesostructures can exceed 3,000 N axial force, even after thermal aging, and are still greater than the maximum posterior masticatory forces reported in the literature, which were in the range of 700-900 N^25^. Therefore, Zr restorations must be able to withstand masticatory forces in the molar areas.

In the present study, the HaC and HC groups had bilayered crowns; however, in the HaC group, the veneering ceramic was cemented over the mesostructure, whereas in the HC group, it was bonded to the mesostructure. The best stress distribution was found in HaC crowns because the cement can act by reducing the stress generated in the covering ceramic, in addition to not having a screw passage hole. The mesostructures proposed in the study were designed to be anatomical, that is, they follow the same pattern as the dental crown. Variations in mesostructure design can lead to differences in the results. Studies in the literature suggest that anatomical mesostructures are more advantageous in terms of restoration strength^25^. The design used in the study helps with the validation of the model since is possible to compare the current FEA results with that of the previous studies using hybrid abutments. However, the best way to validate 3D FEA models is to conduct in vitro and in vivo experimental studies simultaneously^28^.

In the present study, it can be assumed that all groups tested could withstand the mean physiological occlusal forces in the posterior region. Besides FEA is a powerful numerical technique for simulating the behavior of complex structures and materials, but it also has its limitations. The present study used a static analysis with a linear contact type. Exploring other factors, for example, mechanical fatigue should be performed to explain the differences between the results of the present study and those observed in the literature. Long-term clinical studies are also needed to better understand and validate the results presented in this study and others in the literature, particularly on hybrid ceramic materials, which do not present longevity data.

Care must be taken when deciding on the best possible restorative solution for each patient, considering occlusion, parafunctional habits, implant position, esthetics, cost, and degree of complexity. Within the scope of an in-silico study, the conclusions of the study are, mesostructures made of materials with a higher modulus of elasticity exhibit a higher concentration of stress, the presence of a screw hole increases the stress concentration in the ceramic crown and cemented ceramic crowns have better biomechanical behavior than screw-retained crowns.
